# Expression of Multidrug Resistance Genes in Peripheral Blood of Patients with Refractory Epilepsy and the Reverse Effect of Oxcarbazepine on Its Expression

**Published:** 2018-01

**Authors:** Jinming JI, Gang LI, Yunxia MA, Shuangshuang PAN, Rongrong YUAN

**Affiliations:** 1. Dept. of Neurology, Binzhou People’s Hospital, Binzhou, Shandong, China; 2. Dept. of Neurology, Binzhou City Center Hospital, Binzhou, Shandong, China

**Keywords:** Refractory epilepsy, Multidrug resistance gene, Multidrug resistance-associated protein 1

## Abstract

**Background:**

We aimed to investigate the expression levels of multidrug resistance gene 1 (MDR1), multidrug resistance-associated protein 1 (MRP1) and multidrug resistance P-glycoprotein (P-gp) in peripheral blood of patients with refractory epilepsy.

**Methods:**

Patients with epilepsy (n=24) and those with refractory epilepsy (n=24) were selected, and 30 normal volunteers were enrolled as control. The expression level of MDR1 genes was detected using semi-quantitative reverse transcription-polymerase chain reaction (RT-PCR). The expression levels of P-gp and MRP1 were detected via Western blotting. The above-mentioned patients with refractory epilepsy were randomly divided into the oxcarbazepine group (OB group) and placebo group (OZ group). After consecutive 8-week oral administration of drugs, the curative effect and adverse reactions of patients with refractory epilepsy were observed, and the life quality of patients was evaluated.

**Results:**

The expression levels of MDR1 genes, P-gp and MRP1 in peripheral blood of patients with refractory epilepsy were significantly increased compared with those of patients with epilepsy, (*P*<0.05). At 8 weeks after the drug therapy, the effective rate and life quality of patients in OB group were significantly higher than those of patients in OZ group (*P*<0.01). There was no significant difference in the incidence rate of adverse reactions during the treatment between the two groups. After treatment, the expression levels of MDR1, P-gp and MRP1 in peripheral blood of patients in OB group were significantly lower than those of patients in OZ group (*P*<0.01).

**Conclusion:**

Oxacillipine could effectively improve the effective treatment rate of patients with refractory epilepsy. The mechanism may be related to MDR1, MRP1 and Pgp expression.

## Introduction

Epilepsy is a chronic neurological disease. Standard and systematic treatments will effectively mitigate its symptoms of 80% of the patients, but about 20% of the patients with epilepsy have no sensitivity to antiepileptic drugs, referred to as refractory epilepsy clinically (1, 2). Patients suffer from repetitive harms from refractory epilepsy, and their mentality and family and social safety are threatened. The occurrence of refractory epilepsy mostly depends on the genetic factors, disease factors and social psychological factors (3, 4).

The formation of abnormal synapses between neurons provides the basis for the occurrence of epilepsy. That abnormal neural networks occur in refractory epilepsy, leading to abnormalities in nerve excitability conduction, which forms excitatory loops, thus resulting in significantly higher sensitivity of patients with refractory epilepsy than that of patients with epilepsy (5).

Multidrug resistance gene 1 (MDR1) contributes to the encoding of the expression of drug transporter-multidrug resistance P-glycoprotein (P-gp) as well as the regulation of the multidrug resistance-associated protein 1 (MRP1). The over-expression of MDR1 may have a relationship to the drug resistance of epilepsy (6). Exclusively using oxcarbazepine can not only effectively control epilepsy, but also remarkably alleviate epilepsy that other drug cannot deal with (7).

At present, oxcarbazepine’s efficacy in the treatment of epilepsy is ranked as level A by the US Food and Drug Administration (FDA) (8). The correlation of the expressions of MDR1, P-gp and MRP1 in peripheral blood with the refractory epilepsy, and the regulatory effect of oxcarbazepine on the above proteins have not been studied.

This study aimed to illustrate the mechanism of refractory epilepsy at the molecular level and the curative effect of oxcarbazepine on refractory epilepsy through analyzing the relationship of the expressions of MDR1, P-gp and MRP1 in peripheral blood with refractory epileps, and whether oxcarbazepine could regulate it, thus providing new methods for clinically treating refractory epilepsy.

## Materials and Methods

### Instruments and materials

Oxcarbazepine tablets (Novartis Farma S.p.A, Switzerland); dimethylsulfoxide (DMSO) (Sigma); TRIzol kit (Invitrogen); reverse transcription kit (Invitrogen, USA); ELISA kit (R&D system, USA); electrochemiluminescence (ECL) solution (Invitrogen, USA); rabbit anti-Ppg, rabbit anti-MRP1 and rabbit anti-glyceraldehyde-3-phosphate dehydrogenase (GAPDH) (Cell Signaling Technology); horseradish peroxidase-labeled anti-rabbit secondary antibody (Cell Signaling Technology); polymerase chain reaction (PCR) instrument (Applied Biosystems, USA); ultraviolet imaging system (Biometra, Germany); electronic balance (BP121S, Sartorious, Germany); other relevant equipment and reagents were illustrated in relevant parts.

### Study subjects

All the study samples were from the epilepsy patients enrolled in Binzhou City Center Hospital, Huimin County, Binzhou, Shandong Province, P.R. China from from May 2013 to May 2015 who were the confirmed cases diagnosed by specialists. Patients with epilepsy (n=24) and those with refractory epilepsy (n=24) were selected, and the normal subjects were enrolled as control (n=30). Patients with epilepsy consisted of 11 males and 13 females at the age of 31–43 yr old; patients with refractory epilepsy included 12 males and 12 females aged 29–43 yr old; in control group, subjects consisted of 15 males and 15 females at the age of 30–45 yr old. Age and gender of the selected patients were not statistically different from those of subjects in the control group.

The diagnostic criteria for refractory epilepsy referred to the diagnostic criteria formulated by China. Inclusion criteria: 1) patients diagnosed with epilepsy; 2) patients treated with top-ranking antiepileptic drugs for over 1 year; 3) patients whose seizure frequency of epilepsy remained above 30%. Patients with other consumptive diseases were excluded.

All patients signed the informed consent. The experimental regimen was inspected and approved by the Ethics Committee in out hospital. Clinical and pathological data and treatment regimens of all the patients were complete.

### Grouping

According to the type of epilepsy, the selected patients were divided into epilepsy group (n=24) and refractory epilepsy group (n=24). And normal subjects formed control group (n=30). After the serum was separated, the peripheral blood was drawn and then stored at −80 °C for standby application. Patients with refractory epilepsy were randomly divided into oxcarbazepine group (OB group) and placebo group (OZ group) with 12 patients each. Patients took fundamental antiepileptic drugs when being included in this experiment (No oxcarbazepine and/or similar other drugs), and were treated with the experimental regimen stipulated in the treatment: additionally, patients orally took 150 mg oxcarbazepine in two divided doses with one in the morning and the other one in the evening in OB group; patients underwent oral administration of 100 mg oryzanol tablets in two divided doses with one in the morning and the other one in the evening for 8 successive weeks in OZ group. After the experiment, the serum was separated, and peripheral blood was abstracted and stored at −80°C for standby application.

### Evaluation of the treatment for the selected patients with epilepsy

The onset frequency of epilepsy and the onset of epileptiform discharges of patients shown in the electroencephalogram were assessed at 8 weeks after the epilepsy patients received the treatment with antiepileptic drugs, so as to evaluate, the effective rate of epilepsy treatment. Manifestations of the effective treatment. The onset frequency was significantly reduced, and electroencephalogram revealed that the onset of epileptiform discharges was also decreased. Whether dizziness, rash, nausea and other adverse reactions occurred in patients when they were taking drugs was carefully observed. The internationally recognized inventory of subjective life quality (ISLQ) was applied to evaluate the life quality of patients after drug therapy (10).

### Semi-quantitative RT-PCR was used to detect the expression of MDR1

The cryopreserved peripheral blood in each group was taken. Every 500 μL sample was added with 1mL TRIzol reagent strictly according to the instructions of TRIzol kit, and then the solution was shaken and fully mixed. The centrifugation at 12000rpm at 4 °C for 10min was then conducted. The supernatant was absorbed, transferred into the centrifuge tube and added with 500 μL chloroform, followed by ice bath for 10 min and centrifugation at 12000 rpm at 4 °C for 10 min. Afterwards, the supernatant was transferred into the new centrifuge tube, and the same volume of isopropyl alcohol was added followed by 5 min standing. Furthermore, 75% ethanol was added for washing twice after the centrifugation at 12000 rpm at 4 °C for 10 min. Then after the centrifugation at 12000 rpm at 4 °C for 2 min, the ethanol was removed, the solution was dried at room temperature, and the total RNA was obtained after the dissolution with 100 μL DEPC water. The agarose gel electrophoresis and a microplate reader were applied to determine the quality of RNA, and the results manifested that the RNA had good quality eligible for follow-up experiments. After the addition of the components of reverse transcription, its procedures were strictly set up on the basis of the instructions of reverse transcription kit. Reaction procedures: at 52 °C for 5 min and 72 °C for 20 min. After complementary DNA (cDNA) was gained, semi-quantitative RT-PCR was used for determining the expression level of MDR1 with GAPDH as the internal reference. Reaction conditions: annealing at 95 °C for 5 min, 95 °C for 30 s, 64 °C for 25 s, 72 °C for 30 s, a total of 35 cycles, and then extension at 72 °C for 5 min. After the reaction, agarose gel electrophoresis and observation were conducted using ultraviolet imaging system, and the relative expression level of MDR1 in each group was detected with the band gray scale of MDR1/GAPDH as the result. Primers were synthesized by Tiangen Biotech Co., Ltd., and sequences are shown in Table 1.

**Table 1:** PCR primers

	**Sequence**
MDR1	Forward primer: 5′-CAAGATCCTCCTGCTGGATGA-3′
	Reverse primer: 5′-GAACCACTGTTCGCTTTCTC-3′
GAPDH	Forward primer: 5′-GGAAGGTGAAGGTCGGAGTC-3′
	Reverse primer: 5′-CGTTCTCAGCCTGACGGT-3′

### Western blotting measured the expression level of related proteins

The sample of each group was taken and added with radioimmunoprecipitation assay (RIPA) lysis buffer (RIPA:phenylmethylsulfonyl fluorid (PMSF)=100:1) for mixing and splitting for 10 min. After centrifugation at 12000 rpm at 4 °C for 15 min in the centrifugal machine, the supernatant was removed, and BCA protein quantification kit (Themo Fisher Scientific, USA) was used to quantify proteins, so as to prepare protein loading sample at the equal concentration. SDS-PAGE was conducted with the prepared 10% spacer gel, and then proteins were transferred onto the polyvinylidene difluoride (PVDF) membrane under the constant current of 300A for 90 min. The membranes were immersed in tris-buffered saline with Tween 20 (TBST); the target bands were cut and sealed in 5% skim milk at room temperature for 1.5 h. Then these bands were incubated at 4 °C overnight using P-gp and MRP1 primary antibodies (diluted at 1:1000). After the membrane was washed for three times with TBST, the secondary antibody (diluted at 1:5000 with skim milk) was incubated for 1 h at room temperature; after the membrane was washed with TBST for three times, an appropriate amount of ECL solution (solution A and B were mixed at 1:1) was added in the dark room. After tabletting and film washing via automatic developing machine, the film was dried and scanned, the gray value was calculated using Quantity One software and then the film was stored. The experiment was repeatedly conducted for 3 times.

### Statistical analysis

Data in this study are presented as mean ± standard deviation. Statistical Product and Service Solutions (SPSS) 19.0 software (SPSS Inc., Chicago, IL, USA) was used for data processing. Intergroup comparisons were performed using the *t* test; comparisons of count data were conducted using the chi-square test; analysis of variance was used for comparisons among groups. Homogeneity test of variance showed that if the variance was homogeneous, Bonferroni method was used for pairwise comparisons; otherwise, Welch’s method would be applied. Dunnett’s T3 method was used for multiple comparisons. *P*<0.05 suggested that the difference was statistically significant.

## Results

### Expression of MDR1 in peripheral blood of patients with refractory epilepsy

**Fig. 1:**
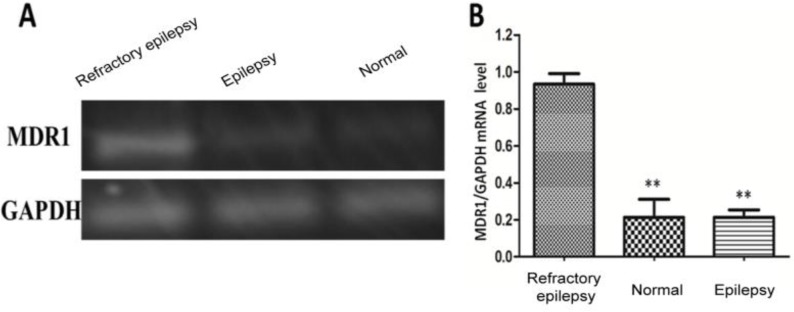
Detection of mRNA expression of MDR1 in peripheral blood via semi-quantitative RT-PCR; A) agarose gel electrophoresis chart, B) statistical chart. The mRNA expression level of MDR1 in peripheral blood of patients with refractory epilepsy is significantly higher than that of normal subjects and epilepsy patients; compared with patients with refractory epilepsy, **P<0.01; there is no statistically significant difference in the expression level of MDR1 in peripheral blood between normal subjects and epilepsy patients (*P*>0.05)

Semi-quantitative RT-PCR was conducted for the detection of the mRNA expression level of MDR1 in peripheral blood of patients with refractory epilepsy. The results (Table 1) showed that the mRNA expression level of MDR1 in peripheral blood of patients with refractory epilepsy was significantly higher than that of normal subjects and patients with general epilepsy, and the differences were statistically significant (*P*<0.01). There was no statistically significant difference in the expression level of MDR1 in peripheral blood between normal subjects and epilepsy patients (Fig. 1).

### Expressions of P-gp and MRP1 in peripheral blood of patients with refractory epilepsy

**Fig. 2:**
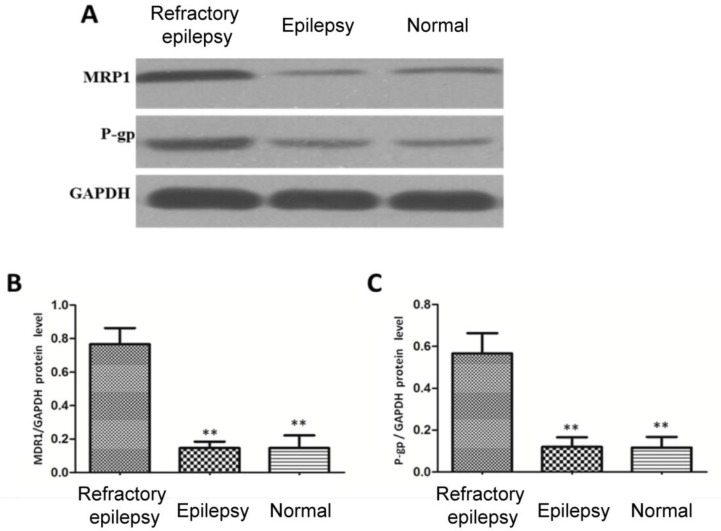
Detection of P-gp and MRP1 expressions in peripheral blood via Western blotting. A) Bar graph, B&C) statistical charts. The expression levels of P-gp and MRP1 in peripheral blood of patients with refractory epilepsy are significantly higher than those of normal subjects and epilepsy patients; compared with patients with refractory epilepsy, **P<0.01; there are no statistically significant differences in the expression levels of MDR1 and P-gp in peripheral blood between normal subjects and epilepsy patients (*P*>0.05)

Western blotting was used to detect the expressions of P-gp and MRP1 in peripheral blood of patients with refractory epilepsy, and its results (Fig. 2) revealed that the expression levels of P-gp and MRP1 in peripheral blood of patients with refractory epilepsy were significantly higher than those in normal subjects and patients with general epilepsy (*P*<0.01). There were no statistically significant differences in the expression levels of MDR1 and P-gp in peripheral blood between normal subjects and epilepsy patients.

### Treatment effect of oxcarbazepine on refractory epilepsy

The effective rate of treatment of refractory epilepsy was evaluated after patients in OB group and OZ group were treated with oxcarbazepine and oryzanol for 8 weeks, respectively, and the life quality of patients was evaluated. The effective rates of treatment are shown in Table 2. The effective rate of treatment was 66.67% in OB group and 8.33% in OZ group, indicating that the effective rate in OB group is significantly higher than that in OZ group (*P*<0.01). The life quality of patients is shown in Table 3. The total score of OB group was significantly higher than that of OZ group; in other words, the life quality of the former was significantly higher than that of the latter (*P*<0.01). During the medication, no serious adverse reactions could be found.

**Table 2:** Effective rate of treatment of refractory epilepsy

**Group**	**Effective rate of drug therapy (%)**	***P***	**χ^2^**
OB group	66.67 (16/24)	<0.01	3.98
OZ group	8.33 (2/24)		

**Table 3:** Life quality of patients with refractory epilepsy before and after treatment

**Group**	**Scale score**		**Interpersonal/social consequences**		**Internal/emotional issue**		**Daily life**	

	**Before treatment**	**After treatment**	**Before treatment**	**After treatment**	**Before treatment**	**After treatment**	**Before treatment**	**After treatment**
OB group	33.5±10.6	59.8±8.6	13.4±2.2	21.6±3.9	10.1±3.2	19.6±2.3	10.2±3.8	18.6±6.5
OZ group	32.6±9.3	41.7±6.3	12.9±3.6	15.7±3.9	10.6±2.5	13.2±1.8	9.8±4.25	11.7±4.9
*P*	>0.05	<0.01	>0.05	<0.01	>0.05	<0.01	>0.05	<0.01
*t*	1.368	0.935	1.258	0.563	0.985	1.025	0.852	0.358

### Effect of oxcarbazepine on the mRNA expression of MDR1 in peripheral blood

**Fig. 3:**
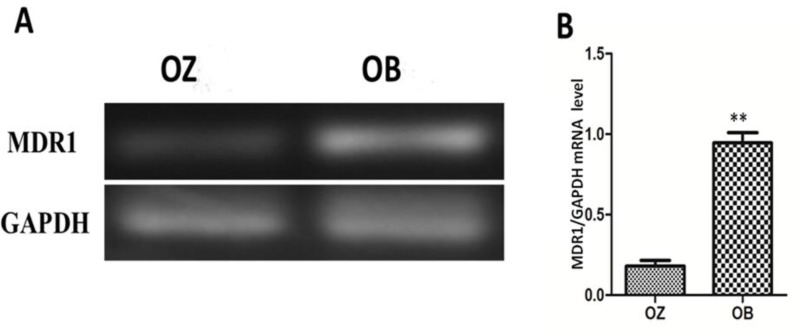
Detection of mRNA expression of MDR1 in peripheral blood after treatment for 8 weeks via semi-quantitative RT-PCR; A) agarose gel electrophoresis chart, B) statistical chart of the relative mRNA expression of MDR1. The results show that the mRNA level of MDR1 in peripheral blood in OB group is significantly higher than that in OZ group (**P<0.01)

At 8 weeks after the treatment with oxcarbazepine, semi-quantitative RT-PCR was used to measure the mRNA expression level of MDR1 in peripheral blood of patients with refractory epilepsy. As shown in Fig. 3, the mRNA level of MDR1 in peripheral blood in OB group was significantly higher than that in OZ group, and the difference was statistically significant (*P*<0.01) (Fig. 3).

### Effects of oxcarbazepine on P-gp and MRP1 expressions in peripheral blood

**Fig. 4:**
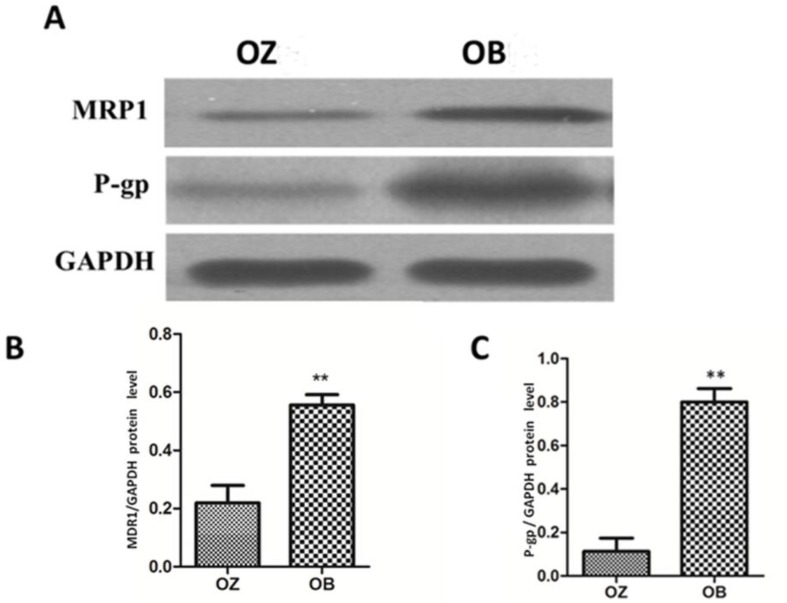
Detection of P-gp and MRP1 expression levels in peripheral blood in patients after treatment for 8 weeks via Western blotting; A) bar graph of Western blotting; B&C) statistical charts of P-gp and MRP1 expressions. The results show that P-gp and MRP1 levels in peripheral blood in OB group are significantly higher than those in OZ group (**P<0.01)

At 8 weeks after the treatment with oxcarbazepine, Western blotting was used to determine the changes in expressions of P-gp and MRP1 in peripheral blood of patients with refractory epilepsy. As shown in Fig. 4, the levels of P-gp and MRP1 in peripheral blood in OB group were significantly higher than those in OZ group, and the differences were statistically significant (*P*<0.01).

## Discussion

Sometimes, refractory epilepsy results in the failure of epilepsy treatment, so that the goal of treating epilepsy cannot be achieved even though the drugs were set in the therapeutic dose in peripheral blood or brain tissues (10, 11). Multidrug transporters are expressed on the cell membrane of brain tissues in patients with refractory epilepsy, which can pump the drugs out of the cells, so the intracellular drug concentration cannot reach the ideal concentration, thus leading to the failure of the epilepsy treatment (12). P-gp and MRP1 are multidrug transporters that are often studied. These two transporters participate in the transport of refractory epilepsy drugs and affect the therapeutic effect of refractory epilepsy (13, 14).

The curative effect of antiepileptic drugs in the treatment of rats with refractory epilepsy can be improved by regulating P-gp and MRP1 in brain tissues of them suggesting that the regulation of multidrug transporters can effectively eliminate the difficulty in treating refractory epilepsy (15). Through establishing a refractory epilepsy rat model, Emich-Widera et al. (14) found that the P-gp expression level on the temporal lobe in the model is 2.7 times higher than that in the normal group, so it is speculated that MDR1 may take part in the formation of refractory epilepsy. A variety of multidrug transporters of tumor drugs are featured with wide distribution, and P-gp and MRP1 are highly expressed in brain tissues of epilepsy patients, suggesting that it may be closely related to the refractory epilepsy (16). However, it is difficult to access to the brain tissue samples, and if the correlation between multidrug resistance of patients with refractory epilepsy and multidrug transporters can be studied by detecting expression levels of these transporters in peripheral blood, the difficulties in developing refractory epilepsy drugs can be alleviated.

In this study, the expression levels of P-gp and MRP1 in peripheral blood of patients with refractory epilepsy, general epilepsy and normal subjects showed that the levels in patients with refractory epilepsy were significantly higher than those in patients with general epilepsy and normal subjects, which are consistent with the findings of previous study (16) about the expression levels of P-gp and MRP1 in brain tissues. The above-mentioned results manifested that there was a consistency between the expression of P-gp and that of MRP1 in peripheral blood and brain tissues, and the correlation between refractory epilepsy and multidrug transporters could be studied through the peripheral blood samples. The expression levels of P-gp and MRP1 are mainly regulated by MDR1, and MDR1 is a gene having a relatively definite association with epilepsy refractory, which shows high expression in brain tissues of patients with refractory epilepsy (16).

In this study, the expression level of MDR1 in peripheral blood of patients with refractory epilepsy was significantly higher than that of normal subjects and patients with general epilepsy. The above results showed that the relationship between the expressions of MDR1, P-gp and MRP1 and the refractory epilepsy can be studied through detecting peripheral blood. But this method still has some limitations: SqRT-PCR evaluates changes of gene expression only at the gene level, he accuracy of which is less than that of pre qPCR and immunohistochemistry methods.

In this study, we further investigated the expression of MDR1 related proteins by Western-blot, and the results were consistent with semi quantitative RT-PCR, which revealed the relationship between MDR1 and intractable epilepsy at gene and protein levels. The recurrence rate of epilepsy could be effectively reduced and the effective rate of treatment of refractory epilepsy could be increased using oxcarbazepine; furthermore, no serious adverse reactions were found during the 8 weeks of mediation. Oxcarbazepine is a new antiepileptic drug on sale, and its single or combined application can be used for the clinical treatment of the generalized seizure of epilepsy. It is the first-line drug for treating epilepsy at present, and it can also treat the epilepsy through blocking the ion channels of nerve cells (17, 18). At 8 weeks after treatment with oxcarbazepine, the mRNA expression level of MDR1 in peripheral blood of patients with refractory epilepsy was significantly decreased, and the expression levels of P-gp and MRP1 were also significantly lower than those in OZ group. Oxcarbazepine increases the effective rate of treatment of refractory epilepsy, which may be associated with the decreased expression of MDR1 mRNA and the regulation of refractory epilepsy-related transport proteins P-gp and MRP1, suggesting that reducing expression levels of multidrug transporters in patients with refractory epilepsy is of great value for increasing the cure rate of refractory epilepsy.

## Conclusion

MDR1, P-gp and MRP1 were highly expressed in patients with refractory epilepsy, and oxcarbazepine can effectively cure the disease, whose possible mechanism is related to the reduced expression of multidrug transporters. The above study results provide new breakthroughs for the clinical therapeutic drugs of refractory epilepsy.

## Ethical considerations

Ethical issues (Including plagiarism, informed consent, misconduct, data fabrication and/or falsification, double publication and/or submission, redundancy, etc.) have been completely observed by the authors.
